# Computed tomography findings in patients with primarily unknown causes of severe or recurrent epistaxis

**DOI:** 10.1371/journal.pone.0220380

**Published:** 2019-08-01

**Authors:** Noel van Horn, Tobias Djamsched Faizy, Michael Hinrich Schoenfeld, Patrick Kohlmann, Gabriel Broocks, Pascal Haag, Jens Fiehler, Christian Richard Habermann, Murat Karul

**Affiliations:** 1 Department of Diagnostic and Interventional Radiology, Marienkrankenhaus, Hamburg, Germany; 2 Department of Diagnostic and Interventional Neuroradiology, University Medical Center Hamburg-Eppendorf, Hamburg, Germany; 3 Department of Otolaryngology, Marienkrankenhaus, Hamburg, Germany; University Magna Graecia of Catanzaro, ITALY

## Abstract

**Objective:**

In addition to rhinoscopy, computed tomography of paranasal sinuses (CT) may be performed on patients with primary unknown cause of severe epistaxis (SE) or recurrent epistaxis (RE) to further assess the potential cause of bleeding. The aim of this study was to evaluate CT findings during the work-up of intractable epistaxis patients.

**Methods:**

6937 patients were treated in our emergency department with acute epistaxis between 2009–2018. 304/6937 patients underwent CT and rhinoscopy due to intractable SE or RE. 33 patients presented with head trauma prior to epistaxis and were excluded from the final analysis. In 271 cases the primary causes of SE (n = 252) or RE (n = 19) remained unknown. Two observers retrospectively evaluated CT scans for potential sources of epistaxis. Disagreement was settled by consensus. CT and rhinoscopy findings were compared.

**Results:**

In 247/271 (91.1%) SE patients no related pathology was found on CT. A possible cause for epistaxis was found in all RE patients, but only in 5/252 (1.9%) patients with SE.

Most tumours (10/11) and inflammatory conditions (9/10) were found in patients with RE.

In three SE cases, a tumour was suspected on CT, from which two suspicions were refuted during rhinoscopy. CT revealed 10 cases of inflammatory conditions of the sinus and anatomical variant as potential cause of bleeding.

**Conclusion:**

For patients with unknown causes of epistaxis, supplementary CT imaging may be a useful diagnostic add-on to rhinoscopy in the event of RE, tumour suspicion or inflammation of the paranasal sinuses. However, in most cases of first-time SE, CT does not necessarily add to the diagnosis. In these cases, the marginal benefit of CT needs to be weighed carefully against its risks.

## Introduction

Nearly 60% of the population worldwide experience a nosebleed at least once in their lifetime [1]. Although no detailed epidemiological studies exist, approximately 6–10% of all nosebleed patients require medical help in an emergency department in Germany [1–3]. A retrospective study conducted in the United States of America reported 1 to 2 out of 200 visits in the emergency department are due to epistaxis and about 5% of the patients are admitted for inpatient care [4]. Beck et al recently described the varying causes of epistaxis, ranging from digital manipulation, over traumatic or tumour genesis to drug-related or inflammatory causes. Furthermore, they stated, most mild episodes of epistaxis originate from an anterior bleeding source in the nasal cavity, namely the Kiesselbachs’s locus or its related blood supplying arteries. This brought them to the conclusion, if medical attention is needed, most cases of mild epistaxis may successfully be treated in the emergency department by a primary care physician or otolaryngologist during anterior nasal endoscopy, without the necessity of any further diagnostic or therapeutical measures [3]. In contrast, some cases of epistaxis can be intractable, e.g. in form of severe or recurrent bleedings. Severe bleedings originate more frequently from posterior arteries inside the nasal cavity (e.g. branches of the sphenopalatine artery or the posterior ethmoidal artery) and due to their anatomical localizations, they may not be approachable for haemostatic primary medical care measures or anterior nasal endoscopy [5, 6]. Accordingly, further assessments of the bleeding source need to be considered, especially if no obvious causes for epistaxis, such as conditions of severe head trauma, are evident. However, no uniform guidelines exist for diagnostic and therapeutic procedures in patients with epistaxis [3]. Therefore, the adequate treatment of patients suffering from intractable epistaxis remains a matter of individual case-sensitive decision making by an otolaryngologist [5, 7, 8]. Rhinoscopy or endovascular digital subtraction angiography (DSA) are invasive procedures by nature but provide both, diagnostic and therapeutical possibilities at one time [9, 10]. Non-invasive diagnostic imaging approaches, such as non-contrast computed tomography (CT) of the paranasal sinuses are usually not necessary [3], but may be conducted in order to detect primarily unknown causes of the bleeding, e.g. neoplastic diseases [11, 12]. Yet, no studies exist investigating CT imaging findings in patients with unknown causes of severe epistaxis (SE) or recurrent epistaxis (RE). In this retrospective study, we investigated CT imaging findings in a large cohort of patients with intractable SE or RE. We investigated, if diagnostic CT supplementary to rhinoscopy or DSA may be a useful add-on for the diagnostic work-up of epistaxis bleeding sources and evaluated to what extent CT findings match or differ from findings of rhinoscopy.

## Methods

### Inclusion criteria

In this retrospective study, all patients with epistaxis that required medical attention in the emergency department of our tertiary care hospital between January 2009 and January 2018 were analysed. The study protocol was approved by the local ethic committee (Ethic Committee of the University Medical Center Hamburg-Eppendorf, Germany), and informed consent was waived.

We identified a total number of 6937 epistaxis cases in our clinical database. Most of the patients underwent successful treatment in the emergency department by a primary care physician or otolaryngologist, without the need for further diagnostic imaging, invasive treatment or medical surveillance. These patients were released from the emergency department a few hours after first admission. In contrast, a total number of 304 out of 6937 cases (4.4%) were identified with intractable epistaxis, defined as severe or recurrent epistaxis. All of these patients have been futilely treated with baseline measures in the emergency department and were crossed over to the otolaryngology service for further assessment. Based on clinical measures and the degree of bleeding, the attending otolaryngologist decided on whether to perform a CT in addition to rhinoscopy. After patients’ admission, CT imaging was only performed in the event that: 1) the source of bleeding remained unknown after anterior nasal endoscopy and 2) conservative therapy with nasal packing was not able to stop the bleeding. All 304 patients received supplementary CT imaging in addition to rhinoscopy. A subset of 33/304 patients presented with severe head trauma prior to the bleeding and thus were referred to as cases with apparent cause for epistaxis and excluded from the final analysis. The other 271/304 cases showed no obvious cause for intractable epistaxis and were therefore referred to as unknown causes of epistaxis.

### Definition of severe and recurrent epistaxis

Patients were regarded as suffering from “severe epistaxis”, when they presented with symptoms of epistaxis in the emergency department for the first time and subsequent “first-pass” primary medical care was unable to successfully stop the bleeding. These measures comprised: compression of the nostrils, bringing the patient in an upright position, lowering blood pressure if applicable, and anterior nasal endoscopy performed by an otolaryngologist. Patients were declared as suffering from “recurrent epistaxis” when presenting with severe de-novo epistaxis after a symptom-free interval of at least two months, whilst having been treated with epistaxis in the emergency department before. Consequently, 19/271 patients (7%) were identified suffering from RE, while 252/271 patients (93%) were regarded as SE. All RE and SE patients (except trauma cases) were regarded as cases of unknown epistaxis.

### Patients´ characteristics

Table 1 displays patients´ characteristics, applied radiation dose and clinical measures of the study cohort. Furthermore, haemoglobin values, presence of hypertension and intake of anticoagulants at the time of clinical admission were evaluated as clinical parameters. Hypertension was defined as initial systolic blood pressures at least >130 mmHg and diastolic pressures of at least >80mmHg (as defined by the ACC/AHA committee) [13].

**Table 1:** Patients´ characteristics.

Age (years)	65.9 ± 16.8 (7–97)
Sex (male/female)	164/107
Time from CT imaging to clinical dismissal (days)	6.8 ± 7.2 (2–68)
Dose-length product <DLP> (mGy*cm)	102.1 ± 19.1 (92–132)
Haemoglobin assessed in the emergency department (mg/dl)	13.4 ± 2.3
Use of Anticoagulants	79
Patients with Hypertension	47

Table 1 displays patients´ characteristics of all 271 subjects at the time of clinical admission. Values are displayed as mean ± SD and range (min-max) or absolute numbers if applicable.

*defined systolic blood pressures of >130mmHg and diastolic pressures of >80mmHg.

### Technical setup

All CT images from the investigated time-period were acquired using the same computed tomography scanner (Siemens Munich, Germany, Definition AS, 128-row scanner). The CT examination covered a scan region from the frontal sinus to the maxilla (100kV, 100-120mAs, transaxial field of view 150mm, no gap, collimation 2mm x 128mm x 0.6mm, pitch 0.985, gantry rotation time 1sec and automatic exposure control). Transaxial, sagittal, and coronal images were reconstructed from isotropic voxels with a slice thickness of 2mm.

### Image analysis

Two radiologists (N.v.H. and T.D.F) with 3 and 6 years of experience in head and neck imaging respectively, were blinded to both the findings/written reports of all past CT examinations and all clinical parameters. Images were assessed on a diagnostic workstation. Both investigators re-evaluated all CT examinations and monitored the correspondent CT images for potential structural causes of SE/RE, namely tumours, acute fractures, inflammatory processes and anatomical variants. In case of disagreement between the two readers, a settlement was reached during a final reading of both readers together. Solely in n = 7 cases, the diagnosis did not coincide, so that a consensus was found during a joint reading.

The final evaluation was twofold: In a first step, all CT findings acquired during the retrospective re-evaluation by both readers were compared to past CT findings as recorded in the written reports. Secondly, respective findings of the CT re-evaluation were compared and juxtaposed to findings of rhinoscopy.

### Rhinoscopy and invasive treatment

Rhinoscopy was performed either at the same day of clinical admission or not later than the day after clinical admission. Rhinoscopy for cauterization or ligation of the bleeding origin was performed under general anaesthesia. In 12/271 cases (4.4%), haemostasis of epistaxis was not achieved by nasal packing and/or consecutive rhinoscopic cauterization (i.e. after removal of the nasal packings) and patients were crossed over to endovascular particle embolization performed by an interventional neuroradiologist using DSA.

### Statistical analysis

Data are presented as mean ± standard deviation (SD) and range. For data analysis and spreadsheets SPSS 24.0 (IBM, Armonk, New York, USA), was used.

## Results

### Findings of CT examination

**Fig 1:**
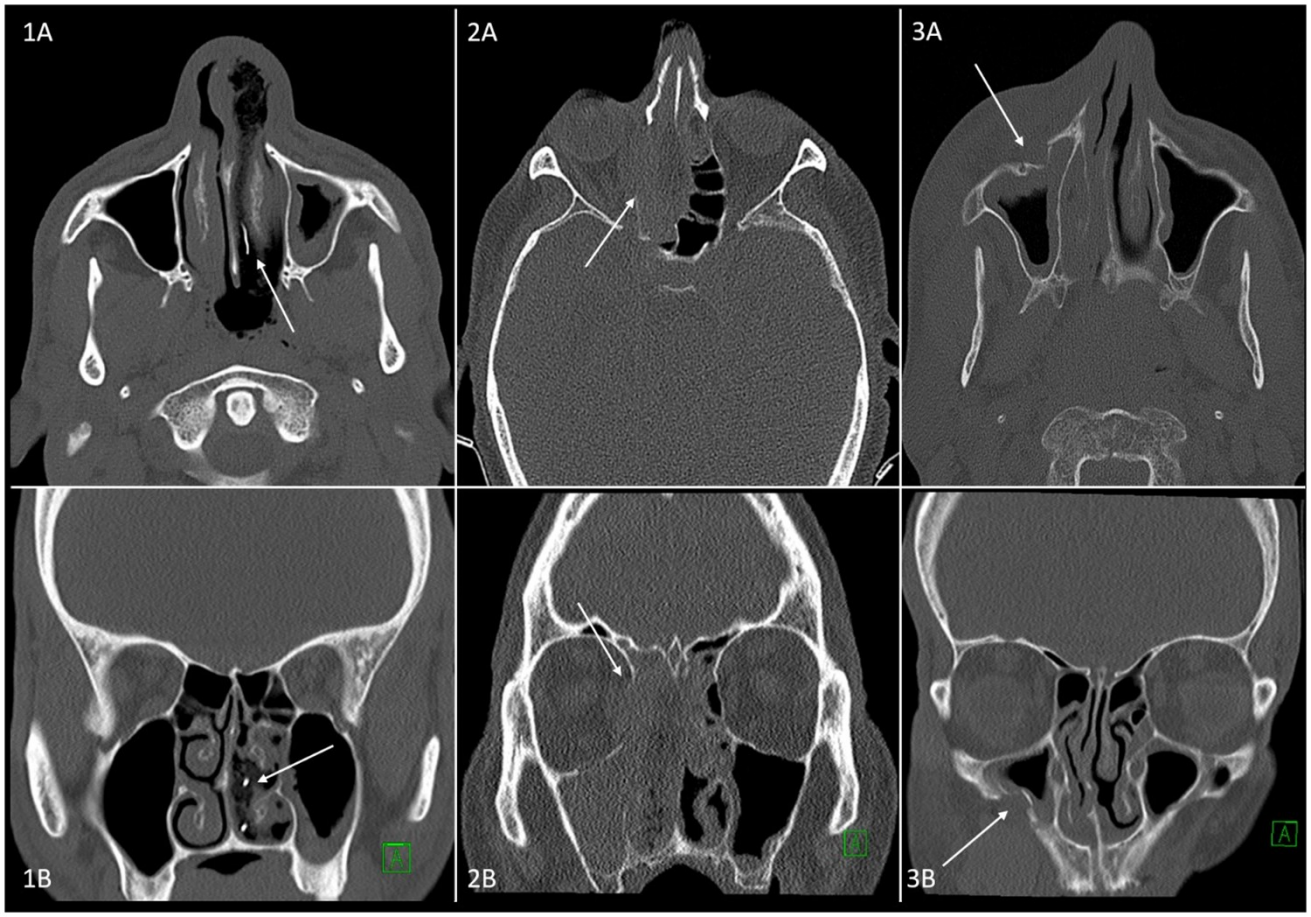
Examples of typical imaging findings in CT. Fig 1 displays examinations of non-contrast computed tomography of paranasal sinuses (CT) of 3 different patients (numbered 1–3), each with axial (upper row) and coronal (lower row) reconstructions. In column 1, white arrows indicate a nasal tamponade in a patient with SE due to bleeding of sphenopalatine artery. In column 2, white arrows indicate a tumorous mass (squamous cell carcinoma) in the nasal cavity, which infiltrates the medial wall of the eye cavity as well as the nasal septum and maxillary sinus. In column 3, arrows indicate a fracture of the anterior wall of the maxillary sinus with consecutive circular swelling of the mucous tissue and epistaxis.

In 33/304 cases, fractures of the nasal bone or paranasal sinuses were detected and suspected as cause of bleeding (excluded cases, see above). Therefore, 271 patients remained with primary unknown cause for intractable epistaxis. Of all these 271 patients, 19/271 patients suffered from RE and 252/271 patients suffered from SE respectively. A number of 10 out of 11 tumours were detected in RE patients on CT images, whereas all 11 tumours were verified in rhinoscopy (see below). Histopathology revealed n = 3 lesions as adenocarcinoma, n = 3 as juvenile angiofibroma and n = 5 lesions as squamous cell carcinoma of the nasal cavity or the paranasal sinuses. In the other 9 RE patients CT showed indications of inflammatory conditions. Fig 1 exemplifies typical findings of CT in 3 different patients. In 247/252 SE patients no related pathology was detected in CT. Suspicion of tumour was found in 3 patients, an inflammatory process in one and one anatomical variant was found to be an extensively huge osseous spur of the nasal septum and was confirmed as bleeding source during rhinoscopy. Table 2 displays a comparison of the systematic CT survey of RE and SE patients, excluding the subgroup of 33 fractures.

**Table 2:** CT findings in patients with RE and SE.

CT diagnosis	RE (n)	(%)	SE (n)	(%)
Normal findings	0	0	247	98
Tumours of paranasal sinuses	10	52,6	3	1,2
Indications of inflammatory processes	9	37,4	1	0,4
Anatomical variants	0	0	1	0.4

Total number of patients included 271 patients with unknown cause of epistaxis: RE n = 19; SE n = 252.

### Findings of rhinoscopy

Table 3 displays the findings of rhinoscopy. From 233 cases with evidence of a direct (out of a related blood vessel) or indirect (tissue bleeding, ulcers etc.) bleeding source, n = 8 patients showed bleeding from the anterior nasal cavity, either from Kiesselbachs´s plexus or originating directly from the septal branch of anterior ethmoidal artery. In 225 patients, bleeding localization was found in the posterior part of the nasal cavity, namely originating from the posterior ethmoidal artery or sphenopalatine artery. In patients with a detectable source of bleeding, consecutive cauterization or artery ligation was performed until haemostasis was induced.

**Table 3:** Rhinoscopy findings in patients with RE or SE.

Rhinoscopy diagnosis	Number of findings (n)	Percentage (%)
Normal findings	16	5.9
Tumours of paranasal sinuses	11	4.1
Indications of inflammatory processes	10	3.7
Anatomical variants	1	0.4
Direct or indirect indicators of bleeding source	233	86

Total number of patients included (with exception of trauma sequelae): n = 271.

### Differences in findings between CT and rhinoscopy

13 image findings were suspicious of a tumour. Eleven of these 13 findings were identified in rhinoscopy (and approved as tumour by histopathology), whereas two CT findings were not detectable during rhinoscopy. Rhinoscopy verified the oversized nasal osseous spur (anatomical variant) as the source of epistaxis in one patient.

### Clinical findings

79/271 patients (29,2%) were under treatment with orally applied anticoagulants due to cardiac disease. 47/271 patients (17,3%) presented with hypertension at the time of epistaxis diagnosis.

## Discussion

This study was conducted to evaluate CT imaging findings in patients with intractable epistaxis of primarily unknown cause, in which first-line medical treatment was not able to staunch the bleeding. All intractable epistaxis patients underwent subsequent rhinoscopy and CT imaging in order to detect the source of bleeding that previously could not be identified by anterior nasal endoscopy. In patients with history of head trauma, fractures of the nasal bone or paranasal sinuses were regarded as evident causes of epistaxis. For these patients, several evidence-based guidelines [14–16] exist that justify the additional utilization of CT and therefore, this group of patients was excluded from our analysis. In the remaining RE and SE patients, the reason for intractable epistaxis primarily remained unknown. In this group, all RE patients showed a relevant pathology on CT images, while CT did not reveal any underlying cause of epistaxis in the group of SE patients in most cases. In two SE patients a tumour was falsely suspected on CT imaging. Rhinoscopy findings revealed the exact anatomical source of bleeding in the majority of patients.

In most intractable epistaxis cases (in which primary conservative medical care or anterior endoscopy measures were futile), rhinoscopy can be considered as first step examination in order to better direct any subsequent optional radiological imaging [17]. A clinical practice guideline for epistaxis is currently in development [18], but to date, comprehensive studies on imaging outcomes of epistaxis, as well as uniform diagnostic and therapeutic guidelines for epistaxis are missing [3]. While CT of the paranasal sinuses may be a valuable additional imaging method for the rule out of some diseases [14], it may not be applicable for the routine work-up of intractable epistaxis in many clinical environments [19]. In contrast, the superiority of rhinoscopy has been discussed before and CT imaging is already not considered as an essential passage in order to find the correct diagnosis [3, 17].

In our clinic, CT is regularly performed as a additional procedure in epistaxis patients with an unknown cause of intractable bleeding prior to any attempt of surgical haemostasis. This is done in order to differentiate between different bleeding origins and to obtain specific anatomical information prior to surgery. In the event that rhinoscopy needs to be extended to an ethmoidectomy, knowledge of certain anatomical landmarks, such as the depth of the skull base, the depth of the olfactory fossa (Keros classification), the course of the intracranial carotid artery and the optic nerves is necessary in order to perform a safe procedure.

The potential benefits of additional imaging via CT have to be weighed carefully against the intrinsic risks of the procedure, resulting from the ionising radiation. Early and late effects of radiation are often classified as deterministic or stochastic effects with a bandwidth from mild to severe reactions. Even comparatively low radiation doses (as such from CT of the paranasal sinuses) hold the risk of deterministic effects on the irradiated lenses, which may e.g. potentially cause radiation-induced posterior subcapsular cataracts [20]. Furthermore, the effects of radiation can cause damage to the molecules carrying genetic information leading to an increased probability of cancer and genetic alterations [21]. The youngest patient included into our analysis was 7 years old. Consequently, we would like to point out that juvenile tissue is most receptive for ionizing radiation and most vulnerable for malignant degeneration [22]. In fact, 2% of all malignant tumours worldwide are induced by medical radiation exposure [22], which can be avoided by considering aforementioned risk factors. However, other diagnostic approaches for the assessment of the paranasal sinuses exist, such as magnetic resonance imaging (MRI), which may be an alternative option for children and young adolescents. As we evaluated an extensive number of RE and SE patients, CT examinations showed no evidence of disease in over 90% of the cases. Due to the large number of negative CT findings in patients with no previous head trauma, it may be worthwhile to consider the performance of MRI instead of CT if applicable, since it holds no ionizing radiation. Moreover, in patients with tumour suspicion, contrast-enhanced MRI should be considered as additional imaging modality, due to its intrinsically high spatial resolution and superior delineation of anatomical structures. Our findings suggest that the decision whether to perform additional CT imaging or not belongs in the hand of an experienced otolaryngologist, who thoroughly needs to consider the benefits of medical imaging supplementary to rhinoscopy, in order to prevent unnecessary radiation. Noteworthy, the predominant number of pathological imaging findings were found in patients with apparent causes of epistaxis and were related to trauma sequalae, namely fractures of the paranasal sinuses or the nasal bone. These cases were not further elucidated in our investigation, but, since CT is also capable to detect trauma sequelae, not only of the paranasal sinuses, but also of adjacent bones forming the midface or portions of the skull base [23, 24], in these terms, it may to be superior to rhinoscopy alone. Moreover, CT has been demonstrated to improve the diagnostic performance, especially when massive arterial bleeding is suspected and PE envisaged [6, 10, 25, 26].

For patients with RE, who suffer from periods of de-novo epistaxis within short time intervals, it may as well be reasonable to perform CT imaging in addition to rhinoscopy. Interestingly, in the subgroup of RE patients, we detected 10 out of 11 tumours, as well as 9 out of 10 inflammatory conditions causative for epistaxis in our study. These findings may suggest that RE occurs more frequently in patients with tumours or inflammatory conditions compared to patients suffering from a single episode of intractable epistaxis and therefore, justifying supplementary medical imaging, such as contrast-enhanced MRI (see above), if no contraindications exist (e.g. contrast media allergies, MRI-incapable pacemakers etc.).

In accordance with our findings several studies reported on patients with recurrent episodes of unilateral or bilateral epistaxis, indicating sinonasal malignancies and inflammatory processes, respectively [17, 27–29]. Furthermore, a previous meta-review [30] discovered that radiological imaging of epistaxis patients may be useful to exclude fractures or infections and moreover for the detection of tumours of the paranasal sinus. It is plausible that in case radiological findings suggest the presence of a tumour of the paranasal sinuses, knowledge about its distinct localization, morphology, as well as its anatomical relationship to adjacent structures are urgently needed prior to additional treatment measures. In addition, CT is the gold standard imaging method for recurrent and chronic rhinosinusitis and associated osseous abnormalities of the paranasal sinuses [29]. A study on 150 patients with chronic rhinosinusitis [31] reported of a relevant number of osseous anatomical variants, e.g. such as the presence of concha bullosa or posterior nasal septal deviations. The knowledge of these abnormalities, although not directly related to the bleeding source, may also be important to the otolaryngologist.

On the other hand, the additional benefits from CT imaging in SE patients were less obvious in our investigation. Only a very small number of relevant pathologies were detected on CT imaging, namely one case of an inflammatory process and three cases with tumorous masses (from which two were even misdiagnosed). Consequently, no related pathology was found on 98% of the CT images of SE patients, once more underlining the necessity of a critical consideration of the benefits and risks of additional/routine CT imaging in intractable epistaxis patients.

Moreover, other predisposing factors for epistaxis, namely conditions such as diabetes mellitus, allergic rhinitis, hypertensive crisis, use of anticoagulants and higher age [7, 30, 32, 33] must be considered prior to imaging. A correlation between epistaxis and the use of anticoagulants has been reported by various previous studies [9, 34–37] and is also associated with RE [38]. Therefore, we presume that these risk factors also should be taken into consideration to decide, whether medical imaging in addition to rhinoscopy is necessary.

In the event that both, conservative and endoscopic therapies fail to stop the bleeding, endovascular PE preceded through DSA may be considered as another treatment option. PE enables selective interruption of the blood supply to the sinonasal area, especially if the bleeding originates from the posterior nasal cavity [26]. In these cases, advanced medical imaging including non-contrast CT and CT angiography depicting the intracranial vessel status may be indicated prior to treatment [25, 26].

Our study holds some limitations: Our dataset of patients represents experiences from a single-center. Although we represent one of the biggest ENT-centers in our region, the lack of uniform guidelines limits the opportunity to compare our findings to other institutions. It would be desirable to match our findings with those of related transregional medical centers, to reveal similarities or dissimilarities in imaging findings and therapeutical procedures of epistaxis patients. Furthermore, the number of RE patients included is comparatively small. More extended analysis including greater number of RE patients are needed to verify the assumption that causal differences exist between SE and RE patients. Therefore, the results of our investigation are to be interpreted in descriptive manner only.

## Conclusion

We present the findings of a comprehensive retrospective descriptive analysis of intractable epistaxis patients, who received CT imaging in addition to rhinoscopy, in which baseline medical treatment, as well as anterior nasal endoscopy failed to staunch the bleeding. While rhinoscopy remains the most important primary measure for the predominant number of intractable epistaxis cases, additional CT assessment may be useful in cases of unclear recurrent bleedings, tumour suspicion, head trauma or inflammatory conditions. Considering the only marginal benefit of CT imaging in patients with first-time SE, risks associated with radiation exposure should be carefully taken into account, when additional imaging is considered for this group of patients.

## Supporting information

S1 Minimal Data Set Patients´ imaging and clinical data. The excel sheet in S1 represents the minimal data set including patients´ imaging and clinical data used for our study. The legend for the data sheet can be found within the excel file.
(XLSX)
